# Dataset on information strategies for energy conservation: A field experiment in India

**DOI:** 10.1016/j.dib.2017.11.084

**Published:** 2017-12-06

**Authors:** Victor L. Chen, Magali A. Delmas, Stephen L. Locke, Amarjeet Singh

**Affiliations:** a UCLA Electrical Engineering, United States; b UCLA Institute of the Environment and Sustainability, United States; c UCLA Anderson School of Management, United States; d Western Kentucky University, United States; e Indraprastha Institute of Information Technology, India

## Abstract

The data presented in this article are related to the research article entitled: “Information strategies for energy conservation: a field experiment in India” (Chen et al., 2017) [1]. The availability of high-resolution electricity data offers benefits to both utilities and consumers to understand the dynamics of energy consumption for example, between billing periods or times of peak demand. However, few public datasets with high-temporal resolution have been available to researchers on electricity use, especially at the appliance-level. This article describes data collected in a residential field experiment for 19 apartments at an Indian faculty housing complex during the period from August 1, 2013 to May 12, 2014. The dataset includes detailed information about electricity consumption. It also includes information on apartment characteristics and hourly weather variation to enable further studies of energy performance. These data can be used by researchers as training datasets to evaluate electricity usage consumption.

**Specifications Table**

**Table:** 

Subject area	*Economics, Engineering, Psychology*
More specific subject area	*Energy, Consumer Behavior, Smart Grid*
Type of data	*Experimental data*
How data was acquired	*The electricity use data was collected at the field site by direct measurement during the period from August 1, 2013 to May 12, 2014. Apartment and household characteristics were collected through a survey that was completed by each participating household at the beginning of the experiment. The weather data was obtained from the weatherundergr*ound.com from the Indira Gandhi International Airport.
Data format	*Stata .dta files*
Experimental factors	*The dataset includes information about electricity consumption, weather, apartment, and household characteristics.*
Experimental features	*Aggregate electricity consumption was measured for each participating household. The sampling interval for the high-frequency data is every thirty seconds but has been aggregated to fifteen minute, thirty minute, hourly, and daily measurements. The weather variables update hourly from station measurements.*
Data source location	*Field Site: Indraprastha Institute of Information Technology faculty housing apartments, New Delhi, India*
Data accessibility	*Data is available at*https://dataverse.harvard.edu/dataset.xhtml?persistentId=doi:10.7910/DVN/7MEXN4

**Value of the data**

• Features household high-frequency electricity data consumption at 15 min intervals for 10 months.
• Data among the highest resolution available to-date in a behavioral experiment in a developing country.
• Measures behavioral responses to information about electricity consumption.

## 1 Data

The data described in this article was acquired 24 h a day in a field experiment at Indraprastha Institute of Information Technology in New Delhi, India during the period from August 1, 2013 to May 12, 2014. The data are related to the research article entitled: “Information Strategies for Energy Conservation: A Field Experimentenergy conservation: a field experiment in India”(Victor L. Chen, Magali A. Delmas, Stephen L. Locke, Amarjeet Singh, 2017). [(Chen et al., 2017) [1]. The raw data includes (i) time stamp; (ii) electricity consumption in kilowatt-hour (kWh) per unit time; (iii) weather data; (iv) engagement with the treatment messages and online energy dashboard; and (v) apartment dwelling and occupancy characteristics, which do not vary with time during data collection. For convenience, the data is provided in panel format for time series analysis. For time varying variables, each successive row represents a 15 min, 30 min, hour, or daily increment.

## 2 Materials and methods

The baseline period for all metered apartments was approximately lasted from August 1, 2013 to February 18, 2014. During this baseline period, no behavioral interventions were performed. The treatment interventions began on February 19, 2014 and the treatment groups were sent weekly emails about their electricity usage and were given access to an online electricity dashboard where they could monitor their electricity usage in real time.

## 3 File structure

A Stata.dta file (version 12) for each table has been included. If a table uses data measured at different frequencies or includes a different number of observations, a file has been created for each table and column. For example, Table_2.dta can be used to replicate all of Table 2 while Table_6_1.dta can be used to replicate column 1 of Table 6. A corresponding Stata.do file has been included that will load the appropriate dataset and replicate the specified table.

### 3.1 Variable descriptions

Table 1 describes the variables in each dataset. The frequency of data can be determined by looking at the timestamp.

**Table 1:** Description of variables measured during the experiment.

**Variable**	**Description**
adults	Number of adults in the apartment
apt	Apartment identifier (1–19)
control	Apartment is in the control group
dayofweek	Day of the week
emailsopened	Number of treatment emails that were opened by each apartment
energy	kWh per unit of time
financial	Apartment is in the financial group
finpost	Financial group × Post
floor	Floor on which apartment is located
ga_fin_1	# of sessions × financial group × post
ga_health_1	# of sessions × health group × post
group	Equal to one if the apartment is in the control group, equal to two if the apartment is in the financial group, and equal to three if the apartment is in the health group.
group_name	Group to which each apartment belongs
haskids	At least one child lives in the apartment
health	Apartment is in the health group
healthpost	Health group × Post
hour	Hour of day
hour_temp	Hourly temperature
lnenergy	Natural log of energy per unit of time. Check timestamp for frequency of data.
male	Head of household is male
mc_fin	Emails opened × financial group × post
mc_health	Emails opened × health group × post
mean_daily_temp	Average daily temperature
numberofsessions	Number of times each apartment viewed their online energy dashboard
pctmissing	% of hourly observations missing
post	Equal to one after the treatment began, equal to zero otherwise
timestamp	The time stamp in YYYY-MM-DD hh:mm:ss
tt	Day since beginning of experiment
tt2	tt squared
tt3	tt cubed
uniqueapt	A variable equal to one once for each apartment in the study.
week	Week of experiment
