# Supplementary material for: Dataset on information strategies for energy conservation: A field experiment in India
Source: Data Brief. 2017 Dec 6;16:713–6. doi: 10.1016/j.dib.2017.11.084 (PMC5735253; doi:10.1016/j.dib.2017.11.084)
Supplement: Supplementary file 1 — Supplementary material [file mmc1.docx]

The authors do not have any conflict of interest to declare.

Victor L. Chen,^^[[1]](#footnote-1)^^ Magali A. Delmas,^^[[2]](#footnote-2)^^ Stephen L. Locke,^^[[3]](#footnote-3)^^ Amarjeet Singh^^[[4]](#footnote-4)^^

1. Department of Engineering, UCLA, 7400 Boelter Hall, Los Angeles, CA 90095, USA. E-mail: victor.l.chen@gmail.com. [↑](#footnote-ref-1)
2. Corresponding author. Institute of the Environment and Sustainability & Anderson School of Management, UCLA, La Kretz Hall, Suite 300, Los Angeles, CA 90005, USA. Tel: 310-825-9310. Fax: 310-825-9663. E-mail: delmas@ucla.edu. [↑](#footnote-ref-2)
3. Department of Economics, Western Kentucky University, Bowling Green, KY 42101, United States. E-mail: stephen.locke@wku.edu. [↑](#footnote-ref-3)
4. Indraprastha Institute of Information Technology, B-302, Academic Block, Okhla Industrial Estate, Phase-III, New Delhi 110020, India. E-mail: amarjeet@iiitd.ac.in. [↑](#footnote-ref-4)
